# New learnings on drivers of men’s physical and/or sexual violence against their female partners, and women’s experiences of this, and the implications for prevention interventions

**DOI:** 10.1080/16549716.2020.1739845

**Published:** 2020-03-23

**Authors:** Andrew Gibbs, Kristin Dunkle, Leane Ramsoomar, Samantha Willan, Nwabisa Jama Shai, Sangeeta Chatterji, Ruchira Naved, Rachel Jewkes

**Affiliations:** a Gender and Health Research Unit, South African Medical Research Council, Pretoria, South Africa; b Centre for Rural Health, School of Nursing and Public Health, University of KwaZulu-Natal, Durban, South Africa; c Johns Hopkins School of Public Health, Johns Hopkins University, Baltimore, MD, USA; d Health Systems and Population Studies Division, Icddr,b, Dhaka, Bangladesh; e Office of the Executive Scientist, South African Medical Research Council, Cape Town, South Africa

**Keywords:** Intimate partner violence, violence against women, ecological model, masculinities, prevention

## Abstract

**Background**: Understanding the drivers of intimate partner violence (IPV), perpetrated by men and experienced by women, is a critical task for developing effective prevention programmes.

**Objectives**: To provide a comprehensive assessment of the drivers of IPV.

**Methods**: A comprehensive review of the drivers of IPV, at the end of a six-year programme of research through the *What Works to Prevent Violence Against Women and Girls Global Programme* with reference to other important research in the field.

**Results**: Broadly, we argue that IPV is driven by poverty, patriarchal privilege, and the normative use of violence in interpersonal relationships. These factors also increase childhood trauma, poor mental health and substance misuse, and poor communication and conflict in relationships, which in turn impact on IPV. Disability status, and contexts of armed conflict, or post-conflict, further reinforce and exacerbate these risks. We move beyond describing associations towards describing the causal pathways through which these factors operate to increase IPV.

**Conclusions**: Specific recommendations about the future of further research on drivers of IPV include a greater focus on understanding the causal pathways from drivers to IPV and clearly delineating association from causality in studies, particularly for women and girls with disabilities, in armed conflicts, and adolescent girls and young women. To achieve this, we recommend extensive in-depth qualitative research, and complex quantitative modeling studies. Understanding drivers and causal pathways better will enable the identification of points of entry for the development of more effective IPV prevention interventions.

## Background

A critical focus of the past 25 years of research within the violence against women (VAW) field has been developing understandings of the drivers of men’s perpetration of physical and/or sexual violence against their female partners (intimate partner violence [IPV]), and the risk factors for women’s experiences of this. Such knowledge is foundational for the development of effective IPV prevention interventions, which are required for governments to meet their obligations under the Sustainable Development Goals (SDGs) to eliminate VAWG, including achieving Gender Equality (SDG 5) and advancing Peace, Justice and Strong Institutions (SDG 16), as well as achieving women’s basic human rights and health.

Since the early 1990s there have been significant strides in identifying the drivers of IPV, primarily focusing on ‘risk factors’: that is identifying individual measurable constructs at multiple-levels, which increase women’s risk for, and men’s perpetration of, IPV (e.g. [1,2]). A small companion area of work has used ethnographic and other qualitative methods to understand how social and structural aspects of people’s lives operate to influence this (e.g. [3,4]).

In 1998 Heise [5], drawing on Belsky’s ecological model, argued that risk factors operate at multiple levels – individual, relationship, community and societal – to increase IPV. Over time this has become a dominant approach to understanding the multi-level influences shaping IPV. In 2011, Heise [6] updated the ecological model emphasizing developmental histories (i.e. experiences in childhood) of the male and female partner in mixed-gender partnerships. She also included the ‘conflict arena’, namely the potential immediate triggers of conflict (e.g. alcohol, distribution of household tasks).

Simultaneously an alternative approach to understanding the drivers of violence was developed, which sought to outline how important latent constructs, which are theoretical and cannot be measured directly – such as ideals of masculinity – intersected and operated to impact on IPV. Such an analysis was presented in Jewkes’ 2002 article, which acknowledged the contextual importance of poverty, but also described two ideological positions which fundamentally drove violence – ‘male superiority’ and the ‘culture of violence’ [7] which were both latent constructs. This work described how many of the risk factors commonly measured were manifestations of these underlying constructs (e.g. male superiority, culture of violence), or ‘processes’ (how they operated to drive IPV, e.g. relationship conflict and enforcement of hierarchy). In addition, Jewkes [7] identified a range of ‘influencing factors’ which flowed from and/or impacted these manifestations and processes (e.g. heavy alcohol consumption, women’s low education levels).

In 2014 Fulu and Heise [8] reviewed the evidence-base on the drivers of IPV to provide a foundation for developing further knowledge on IPV prevention. Their review focused on current debates in the field, specifically on how drivers operated to increase IPV, but concluded that there was still too little known to draw definitive conclusions, and that the comparatively little research on men’s perpetration of IPV was an important gap in existing knowledge [8].

In this paper we aim to provide an overview of the recent evidence around the drivers of VAW, focusing predominantly on men’s perpetration of physical and/or sexual violence against intimate female partners, and women’s vulnerability to this violence. We also focus on the implications of this evidence for designing evidence-based IPV prevention interventions.

We draw on two bodies of evidence. First, the work produced through the six year *What Works to Prevent Violence Against Women and Girls Global Programme (What Works)* funded by the UK Department for International Development (DfID), which included analyses of drivers of IPV and 15 prevention evaluations. Second, we draw on the wider body of literature that has emerged, particularly publications describing prevention interventions, as well as publications from important datasets such as the United National Multi-Country Study on Men and Violence in Asia and the Pacific (UNMCS) [9]. We describe the state of current knowledge around drivers of VAW, and the implications for future research, and prevention interventions.

This paper is not presented as a systematic review of the entire body of evidence on drivers of IPV, but rather a comprehensive engagement with new knowledge from the field. It chiefly considers men’s violence against their female partners in mixed-gender relationships, and women’s experiences of this, and generally assumes that both partners are cis-gender. It does not engage with the emerging body of research around violence and conflict in same-sex relationships which mostly comes from HICs.

**Figure 1.**
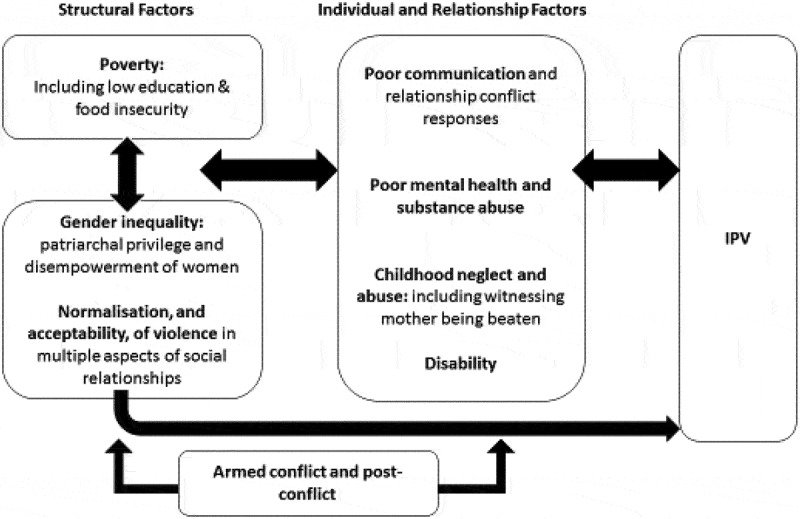
Drivers of IPV

In Figure 1, we provide an updated framework for understanding the drivers of IPV. Underpinning IPV are three structural factors: first, gender inequality in the form of patriarchal privilege and the disempowerment of women, second the normalization, and acceptability, of violence in social relationships, and third poverty. These structural factors individually, and synergistically, drive men’s perpetration of IPV and increase women’s risk of experiencing IPV. They also impact on ‘individual’ level factors, increasing the risk of poor mental health, substance misuse, poor communication and relationship conflict, and childhood abuse and neglect, which in turn also fuel IPV. Disability further exacerbates other risk factors. Armed conflict and the period following conflict operates to further exacerbate the structural factors (gender inequalities, normalization of violence, poverty) as well as ‘individual’ level factors (poor mental health, childhood neglect, disability). While pathways suggest an inevitable relationship between drivers and IPV, experiencing any one (or all) of the drivers, does not necessarily lead to IPV, rather it increases the likelihood it would happen.

## Patriarchal privilege and the disempowerment of women

Jewkes [7] argued the two most important sets of ideas and practices driving VAW were the gender hierarchy within a society, and the extent to which the use of violence was normative in interpersonal relationships. In settings where men’s patriarchal privilege is emphasized (which is everywhere in the current configuration of the gender order), men’s dominance and control over women are normative, and gender attitudes, norms, roles, values, entitlements and identities flow from this [10].

There has been considerable work over the last six years to better understand men’s perpetration of violence, and in particular, to address two key questions: why do men as a group use violence against women? And, why do particular men use violence? Earlier work argued that violence is used by men to express their power over women by punishing real or imagined transgressions, and that the threat of violence itself acts as a means of social control [11]. In this conceptualization, men use violence to assert their dominance and control over women to maintain the gender hierarchy. The work of Raewyn Connell [12,13] has been particularly important in framing understandings of the hegemonic nature of the gender regime within a society. The most powerful form of masculinity, hegemonic masculinity, derives its power in part from the acquiescence of many of those who are subordinated by it, including women and groups of less powerful men [13]. Through this acquiescence, the gender regime within a society can be entrenched without the overt use of force to establish and maintain control. The gender regime also includes formal and informal systems of rewards and sanctions for women who conform or resist further entrenching this control, including legal and policy frameworks. However, the underlying potential of men’s violence against women reinforces the gender regime, and is illustrated through the actions of men who perform more exaggerated masculinities – or hyper-masculinity [14]. Hyper-masculinity visibly deploys physical violence and rape against women [14], and the stress caused by this to those who experience it, or observe it, either directly or indirectly, reinforces the status quo and supports the less overtly violent hegemonic masculinity.

## Why do particular men use violence? Masculinities and the clustering of men’s practices

Research on drivers of violence often fails to show the expected direct connections between measures of individual gender attitudes and IPV perpetration. For example, in the UNMCS dataset, in only two of the six countries were men’s inequitable gender attitudes associated with lifetime IPV perpetration in multi-variable models [1]. This may partly reflect measurement weaknesses and/or operation through indirect pathways.

Another explanation for the lack of observed association between gender attitudes and IPV perpetration is that men’s use of violence may actually be related to how men see themselves as men and their aspirations as men. This may be framed in relation to other men, the family, women, and men’s self-assessment of their success. Further, there may be a disconnect between how men perceive their views on gender and how they position themselves as men and their perceptions of the entitlements that flow from it. In the UNMCS analysis, men’s controlling behaviours in relation to their partners (e.g. always wanting to know their whereabouts) was associated with IPV in all models [1]. Extending this, a structural equation model (SEM) presented in Gibbs et al [15] showed inequitable gender attitudes operated through increasing controlling behaviours to impact IPV perpetration. Thus, it was not just the hierarchical attitudes that were important, but the perceived need to express gendered privilege in dominance and control over women.

Even in contexts which are generally highly patriarchal and lacking great diversity in views on gender equity, such as in many communities in South Africa, there can be a lot of diversity in men’s individual use of violence. Using a population-based sample of South African men, Jewkes and Morrell [16] applied the technique of latent class analysis (LCA) to assess different men’s use of violence. The LCA identified three groups (classes) of men, associated with a clustering of behaviours related to violence and the performance of gender relations in heterosexual masculinities [16]. The most violent men comprised a quarter (24.7%) of the sample and reported high levels of IPV perpetration. A second group of violent men (29.6%) reported slightly less IPV perpetration, and the lower violent men (45.7%) reported very little IPV perpetration [16]. The men in the most violent group expressed an emphasized form of masculinity (or hyper-masculinity), compared to those with the least violence. The most violent category defined through IPV was also strongly associated with non-partner rape perpetration, inequitable gender attitudes and controlling behaviours.

Jewkes and Morrell [16] also assessed what factors were associated with being in the most violent group of men, and revealed the role of deeper poverty, extensive exposure to abuse and neglect in childhood, as well as being bullied in childhood, and, especially for most violent men, having a cruel father [16]. The impact of child abuse and neglect on the psychopathological development of the men has been shown [17] to result in greater instrumentality in relationships, and a limited capacity for remorse, empathy, and a tendency to externalize blame, all of which were measured associations in this study [16]. This analysis demonstrated the interplay between structural factors (poverty), exposure to childhood trauma, individual psychopathology, and the social construction of masculinity, which is informed by ideals of gender positions, aspirations and relations, and how this interaction was in turn associated with individual men’s use of violence.

### Social norms

There is general agreement that ideas about gender and power are socially learnt patterns of thoughts and behaviours that are usually taken for granted and unexamined [18]. Social norms theory distinguishes behavioural patterns (what we, as an individual, do), collective attitudes (what we, as a group, think and feel about something) and individual beliefs about others’ behaviours and attitudes (what we think others would do and think) [18–20]. There has been considerable research showing that the opinions of others matter in relation to behaviour in general, and specifically violence perpetration [18,21]. Social norms theory argues that the views and actions of others matter so much that they strongly influence how we ourselves act.

This understanding of social norms has led to interventions focused on reducing IPV (and violence more widely) through seeking to question the gender structure and the legitimacy given to the use of violence through interventions that essentially challenge gender roles and practices and the acceptability of violence (for example, SASA! [22,23]). There is some evidence of success for interventions using this approach (e.g. [22,24]).

If social norms are as important in driving IPV as the theory implies, we would expect them to be strongly predictive of IPV perpetration, perhaps more so than men’s individual attitudes, and we would also expect change in norms to occur in parallel with, or preceding changes in attitudes. Some *What Works* studies measured individual attitudes and perceptions of community views on gender and gender-related practices, and found that individual attitudes were more strongly predictive of violence perpetration by men than views on what the community thinks (e.g. in Ghana [25]). Furthermore, research on the most violent men suggests that they live their lives in a way that includes great concern for the views of the mainstream (social norms). However, men who are very violent are often strongly influenced by peers, including in extremely violent contexts such as in gangs [4,26]. Additionally, it is well recognized that there is often poor correlation between attitudes and behaviour, and that behaviour change often precedes attitudinal changes [27].

**Figure 2.**
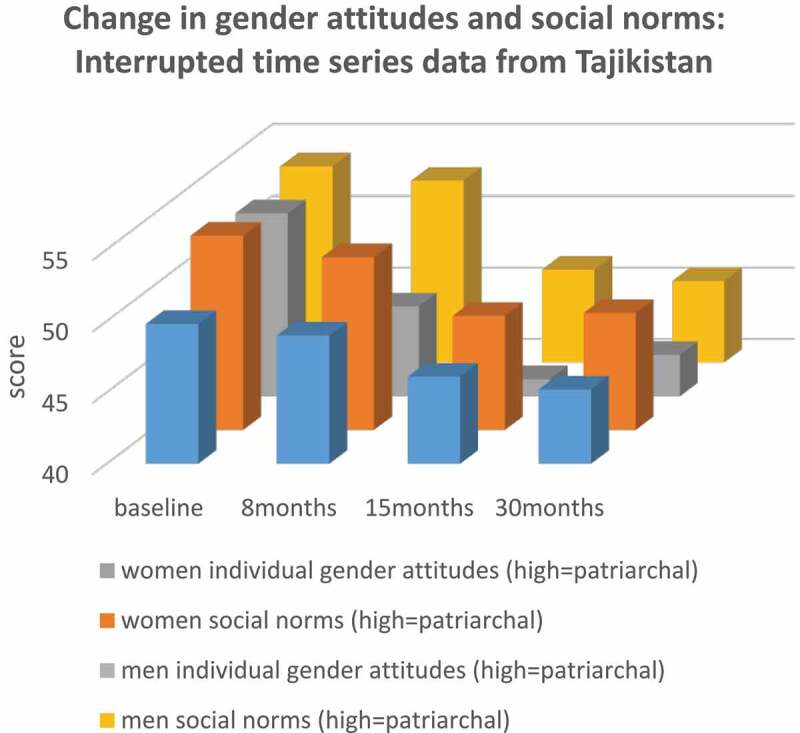
Individual attitudes and social norms in time-series data from Tajikistan

In an evaluation of Zindagii Shoista, a *What Works* study in Tajikistan, which combined family-based economic strengthening and gender transformation over a 15 month period, individual attitudes and social norms were assessed at four time-points [28]. In response to the intervention, women’s and men’s individual gender attitudes became more equitable, and their perceptions of community social norms did likewise (see Figure 2) [28]. The intervention did not include elements that would have changed social norms (e.g. community activism). As such, it is more likely that as women’s and men’s individual gender attitudes changed, they paid more attention to what they heard and saw in the community that was congruent with their own changing ideas, and thus they perceived community social norms to have changed. Thus, perceptions of what others think and do are closely related to what an individual thinks and does in some settings, with the direction of effect being reversed.

The emerging evidence suggests that whilst social norms are important, they vary contextually. Thus, mainstream social norms may not be the most important influencers on individual men, and they are not necessarily the same even for neighbouring communities, and a range of other factors operating in a community influence the importance of social norms on individuals. Furthermore, the impact of social norms is filtered through individual life experience (especially of poverty and trauma), personality, individual attitudes towards gender and violence and so forth. They have a role as expressions of the gender regime within a setting, but they are one of many other drivers of IPV, rather than occupying a privileged position as ‘the’ core driver [29].

## Poverty

One of the key factors which influences men’s violence perpetration and women’s options to leave when exposed to IPV is poverty. There has, however, been debate about the role of poverty as a driver of IPV. Women of all social classes experience IPV and there was concern that the higher levels reported in poorer households was driven by a greater willingness to report IPV. In addition, reviews sometimes show no clear association between markers of poverty and IPV (e.g. assets [30] and employment [31]). There has been, however, considerable growth in the evidence outlining the multiple ways in which poverty is a direct and indirect driver of IPV.

As an indirect driver of IPV, poverty increases the likelihood of experiencing other recognized risk factors for IPV. Growing up in poverty is associated with poorer educational outcomes [32] and less education often leads to less exposure to diverse social norms, and a willingness to challenge social norms. Three *What Works* papers from South Africa, the Asia-Pacific and Afghanistan highlight these associations: in each poverty leads to poorer educational outcomes, which leads to less gender equitable attitudes [15,33,34]. In addition, less education is associated with greater poverty in later life [32].

Growing up in poverty also increases the likelihood of exposure to childhood abuse and neglect, as well as poorer mental health and greater substance use in later life. Studies clearly demonstrate how poverty increases the likelihood of experiencing childhood physical, sexual or emotional abuse, or neglect [33,35], in part because of the challenges of raising children in poverty. Hatcher et al [36] demonstrate that among men living in informal settlements in South Africa, those who grew up in poverty were more likely to experience childhood abuse, and experienced increased depressive symptoms in later life, even after adjusting for experiences of other childhood traumas. Similarly, studies and reviews have shown close associations between childhood poverty and later alcohol and drug use [37–39].

Poverty is also a direct driver of IPV. Studies are fairly consistent in demonstrating that women’s food-insecurity (an indicator of acute poverty) is associated with experience of IPV in both high and low-income settings [1,15,33,40]. While in some studies there is no clear association between poverty and IPV, this may be because the samples often lack sufficient variation in socio-economic status to meaningfully assess the effect (particularly the case in self-selecting samples or samples from one area), or else studies measure multiple indicators of poverty, and treat these as separate constructs, rather than as one latent construct, leading to measured indicators of poverty not being significant in models.

Poverty can also directly increase IPV within relationships. First, in acutely food-insecure households there is likely more stress about the distribution of food (and resources more generally) leading to fights. Second, poor households experience more stress, and struggle more to deal with daily stressors, further increasing conflict. Third, lack of food also has physiological impacts on individuals, leading to less ability to regulate emotions, further contributing to conflict [41,42].

## Childhood experiences of violence and neglect

In contexts of poverty, where patriarchal privilege structures relationships and violence is normative, childhood experiences of physical, sexual and emotional abuse and neglect are much more likely, and children are also more likely to witness IPV. Such experiences in childhood are strongly associated with subsequent IPV experience and perpetration [35,43–45]. There are likely two pathways through which childhood trauma drives IPV: the social learning of violence, and the impacts on children’s brain development and later personality, particularly co-morbid poor mental health and harmful substance use.

A key pathway through which childhood violence (experiencing and witnessing) leads to subsequent IPV is social learning around violence. This mechanism is often referred to as ‘acceptability’ but it is more than that, it is also a process through which the use of violence – when it can be used and what it can achieve – is learned. Social learning theory suggests four mechanisms through which behaviour becomes engrained: observation of others, internalization of attitudes supportive of that behaviour, imitation of role models, and reinforcement of behaviour through rewards and sanctioning [46]. There is much research describing the overlapping nature of violence in households; children who witness their mother being beaten are more likely to perpetrate and experience peer violence [44], and women who experience IPV are more likely to experience violence from other family members [47,48]. Namy et al [49] have described how patriarchal attitudes in family contexts infantilize women and children, enabling the use of violence against both groups.

The second pathway relates to the direct impact of experiences of violence and neglect in childhood on brain development, and later personality, which impacts relationships, and increases poorer mental health and harmful substance use. There is some evidence that the path is partly genetically mediated in suppression of the gene for Monoamine Oxidase A (MAOA) which impacts personality, predisposing individuals to both general anti-social behaviour and a propensity to violence [50]. There is also quite strong evidence that exposure to trauma in childhood impacts on the developing brain, causing long-term changes in brain circuits and systems in response to stress [50]. Children who experience violence in childhood have deep mistrust and insecurity, lack empathy and guilt, and have low self-esteem, which negatively impacts on all relationships [17]. This can draw men towards violent and anti-social peer groups. These experiences can also lead men towards a deep mistrust of women and lack of empathy and guilt, and these have been described in the lives of men who kill their intimate partners [51]. In addition, these changes in the brain structure also increase the likelihood of poor mental health, and harmful substance use.

## Substance use and mental health

Harmful substance use and poor mental health are co-morbid [52] and are both well-established consequences of VAW exposure for women [53,54]. However, they are also key drivers of IPV [53–55], and are themselves often the outcome of poverty, gender inequalities and childhood physical, sexual and emotional abuse and neglect [56].

For women, and men, there is evidence that harmful alcohol is a risk factor for IPV. Devries et al [54] identified three longitudinal studies (all from the US) demonstrating strong associations between binge-drinking and women’s IPV experience. For men, a systematic review of population-based studies found a strong, positive, association between men’s harmful alcohol abuse and recent IPV perpetration [55]. Even where women do not commonly engage in harmful alcohol use, men’s drinking is a risk factor. For example, a four-country study from Asia-Pacific, found if a woman’s partner drank alcohol regularly, they were more likely to experience IPV [33].

There are multiple potential pathways through which alcohol abuse leads to increased IPV. Consuming harmful levels of alcohol can lead to more frequent quarrelling about finances and household responsibilities, as money and time are spent drinking. For couples who often drink together, there may be alcohol-related diminishment of cognitive functioning, increasing the likelihood of arguments in relationships becoming violent. Qualitative research in one IPV prevention trial in South Africa, Stepping Stones and Creating Futures, found that one way women sought to reduce their IPV risk was not arguing with their male partner if he, or she herself, were drunk. While not transforming gender relationships, this was an important harm reduction strategy that emphasized how alcohol, quarrelling and IPV are interlinked [57]. SEMs also demonstrate how alcohol use leads to more quarrelling and then IPV (although what people quarrel about was not specified [15]). In addition, men’s harmful alcohol use can also be part of clustering of other practices, including men’s perpetration of IPV, forming a gender inequitable masculinity [58], reflecting men’s attempts to position themselves as dominant, vis-à-vis their female partners, and other men.

Outside of heavy drug-using populations (e.g. [4,59]), there is little research on the role of substances other than alcohol as a risk factor for IPV, although there is more research around other substances and rape perpetration [26,60]. Cross-sectional studies from *What Works*, have started to show some associations. For instance, among young women [18–30] in urban informal settlements in South Africa, 31% reported past year illegal drug use, and this was associated with increased IPV experience, even after adjusting for alcohol use [43]; the study did not ask about their partner’s drug use. In the four-country UNMCS study from Asia-Pacific, women who reported their partner used drugs also reported more IPV experience, even after controlling for partner alcohol use [33]. In South Africa, a short-intervention among women who used drugs frequently found a reduction in biologically verified drug use by women, but no reduction in IPV [61]. However, when the intervention was adapted and used with couples who both used alcohol and drugs, they saw a reduction in IPV and alcohol and drug use [62], highlighting the role of couple dynamics and male consumption of drugs in IPV.

### Mental health

Women’s and men’s poor mental health is increasingly recognized as a driver of IPV. Among women, a recent systematic review identified six longitudinal studies assessing depression and IPV incidence, with positive associations observed in all studies [53]. Evidence on other forms of poor mental health as drivers of IPV is more limited, and associations have been mainly described cross-sectionally. Furthermore, because poor mental health is both a consequence of, and risk factor for IPV, women often become trapped in a cycle of reciprocal causality, which makes establishing cause and effect more difficult. The mechanisms through which poor mental health increases women’s experiences of IPV are poorly described in the literature. However, it is likely the mechanisms are associated with the underlying drivers of poor mental health (including childhood trauma, poverty) and comorbid drivers (such as substance use), which impact on women’s ability to engage in emotionally connected and trusting relationships.

Men’s poor mental health is associated with IPV perpetration, although the majority of longitudinal studies are from high-income countries, and typically draw on samples comprising US military combat veterans who often have very poor mental health. These studies show strong associations between PTSD and subsequent IPV perpetration [63]. Among other reasons, PTSD may function to increase IPV perpetration by increasing hyper-arousal, and this is associated with dysfunctional responses in relationships and the use of IPV [63]. Other measures of poor mental health show less clear relationships to men’s IPV perpetration. In a nine-country study from Asia-Pacific, depression was associated with men reporting perpetrating both physical and sexual IPV [1]. However, in a systematic review of population-based studies, there was no association between depression and IPV perpetration [55]. Research has also highlighted how men who adhere to more gender inequitable attitudes have worse mental health [64,65], suggesting that poor mental health may also be an outcome of occupying masculine positions which have been linked to IPV perpetration.

Research has tended to separate out harmful substance use and poor mental health as different concepts, as well as focusing on specific manifestations of each (e.g. alcohol use versus drug use, depression versus anxiety), however, these strongly overlap. For women, IPV is a driver of poor mental health and substance misuse, and poor mental health and substance misuse in turn increase women’s risk of experiencing IPV. For men, substance misuse and poor mental health are drivers of IPV. Indeed, the *What Works* funded Violence Alcohol and Treatment intervention (VATU) in Zambia used the Common Elements Treatment Approach (CETA) to tackle symptoms of common mental disorders (depression and anxiety), substance use and IPV, amongst couples where the man had a problem of harmful alcohol use and uses IPV. The RCT evaluation showed significant impacts on reducing IPV, symptoms of common mental disorders and alcohol use [66].

## Disability

According to the WHO, approximately 15% of people in LMICs have a disability [67] and disability is increasingly recognized as an important risk factor for IPV [68,69]. However, it is not yet systematically integrated into IPV prevention research. The majority of *What Works* research projects included the Washington Group Short Set (WGSS) of questions [70] to assess disability and a pooled analysis of *What Works* baseline data demonstrated that disabled women were twice as likely to report recent experience of IPV [71]. Of note is that one of the domains assessed in the WGSS is impairment in cognitive function (remembering/concentrating) which is a common symptom of mental health problems, especially PTSD, which may result from experience of violence.

The pathways through which disability increases IPV risk are likely multifaceted, and bi-directional, but are not yet adequately theorized. There is likely a direct pathway as having a disability can introduce additional stress in the households, through care and support needs, men may resent the care work expected of them, and there may be additional costs. Further, the stigma of disability socially devalues the affected woman and thus reduces her power in the home and community. Women with disabilities may not be able to fulfil ‘traditional’ roles as women, which may expose them to punishment [72]. In addition, disabled women may be more economically and socially dependent on immediate family and caregivers, and therefore face additional barriers in help-seeking and/or trying to exit abusive relationships. Programmes and institutions that serve women experiencing IPV often fail to fully accommodate the access needs of women with disabilities.

There remain many outstanding questions around the association between disability and IPV experience. Many disabilities are not adequately captured in the WGSS, particularly chronic illnesses with intermittent manifestations. In addition, as the majority of research to date is cross-sectional, the extent to which disability drives IPV risk and vice versa is unknown. Further research is also required to understand the pathways through which women living with disabilities have a higher likelihood of experiencing IPV and identify possible points for beneficial intervention.

## Impacts of armed conflict

Armed conflict has the potential to increase the likelihood of IPV, both during the conflict and in its aftermath. Non-partner rape is most closely associated with armed conflict in discussions of conflict-related VAWG, and very often is highly prevalent, yet IPV remains the most common VAWG experience of women living through conflict or in post-conflict settings [73,74]. *What Works* studies in South Sudan and the DRC have highlighted the very high prevalence of VAWG experience in conflict-affected populations: population-based research from South Sudan has shown the lifetime prevalence of non-partner sexual violence experienced by women ranged from 28-33%, while IPV had been experienced by 54–73% of ever-partnered women and girls [75]. Similarly, in the DRC a fifth (20.8%) of women reported non-partner sexual violence in the past year and 68% of women reported experiencing IPV in the same period [75].

The understanding of why armed conflict leads to increased IPV during this time and in its aftermath is only just starting to be theorized. Armed conflict likely has an indirect impact on IPV, through increasing known drivers. Armed conflict and forced displacement increases poverty, through the destruction of livelihoods (crops, property, etc.), and also worsens children’s educational outcomes. Girls, in particular, may be affected as they held back from attending schooling to protect them from conflict-related risks. In addition, the rule of law is eroded, limiting the prosecution of crimes. In South Sudan, for example, the ongoing conflict and lack of rule of war enabled more violent cattle raids, who sought to secure cattle for bride price, but also included rape of women and girls, leading to more community violence and retaliation [76]. Armed conflict also leads to forced displacement and separation from family and support systems, and these may also be potential pathways impacting IPV [75].

Armed conflict, exposure to traumatic events, and the chronic stress of living under constant threat of attack, also worsens mental health and this is often associated with increased substance use. This has been particularly studied among male US combat veterans, where studies have emphasized increased PTSD as a pathway [63] through which trauma exposure increases IPV perpetration. The impact of trauma on poor mental health can last long after conflicts end [77,78].

Armed conflict also impacts on men’s masculinities, in two potential ways. During periods of violence, there may be greater prominence of ‘strong man’ masculinities, fueled by communities wanting protection, but also enabling the normalization and acceptability of violence by men. In contrast, war may lead to the undermining of men’s masculinities, as their livelihoods and positions of authority are destroyed, with men seeking to reassert their power and authority through control of women, including the use of violence if necessary.

These multiple potential pathways through which war conflict increases IPV are described among men in population-based data from Papua New Guinea (PNG) [78]. In men, exposure to war trauma was associated with increased PTSD, and alcohol abuse [78]. While increased IPV perpetration was associated with alcohol abuse, depression and drug use, as well as enduring aspects of war including reduced education, poor employment prospects, and difficulty controlling aggression and feeling unable to trust anyone (which are symptoms of PTSD and anxiety) [78].

While among women, *What Works* analyses have shown potential pathways through which armed conflict increases their experiences of IPV. Among married women in Afghanistan, a SEM showed exposure to war trauma, increased food insecurity, reduced educational outcomes, increased exposure to childhood physical abuse and neglect, and led to less gender-equitable attitudes [34]. In turn, these impacted on poor health (including poor mental health), and led to greater experience of IPV [34]. Similarly, in a non-*What Works* study in PNG for women increased IPV was associated with reporting more enduring impacts of the war, higher depressive symptoms and greater alcohol abuse, which depression and alcohol use were both also associated enduring legacies of the war [78].

These studies suggest that armed conflict increases other known direct drivers of IPV for women and men, rather than being a direct driver of IPV itself. These include entrenching patriarchal social relationships, the normalization of violence, increasing poverty, and worsening mental health and substance use. If correct, these suggest that working collectively around psychological healing and reducing poverty, may be important interventions in communities which have experienced war conflict.

## Implications for understanding drivers of IPV and prevention interventions

The growth in research around drivers of IPV has led to greater understanding of the mechanisms through which drivers and risk factors operate to increase IPV for individuals and populations. Broadly, research needs to move towards developing stronger theoretical understandings of the drivers of IPV and disentangling association and causation, particularly for groups with very high rates of IPV, and use these learnings to develop stronger IPV prevention interventions.

A key task in developing more effective IPV prevention interventions is to disentangle whether measured variables are actual drivers of IPV, or just strongly associated with the ‘actual’ driver (association versus causation). For instance, lack of savings in multivariable models is often associated with IPV experience [79,80]; however, it may be that there is no direct causal pathway between lack of savings and IPV, rather savings may just be an indicator of poverty. Indeed, a number of studies on the impact of Village Savings and Loans Associations (VSLA), and providing girls with access to savings accounts, have shown no impact on experiences of IPV despite increasing their savings [80–83]. As such, it may be that savings is associated with IPV because it is an indicator of poverty, rather than a cause of IPV in and of itself.

Similarly, we need to consider whether measured variables are distinct from each other, or capture aspects of a latent construct (again there are aspects of association versus causation). There are clear overlaps in households between children’s experiences of physical punishment and women’s experience of IPV; this does not however mean that children’s experiences of violence drive IPV in the household, rather they are capturing the underlying latent constructs of the normalization of violence in the household, and male patriarchal privilege.

Our task in developing IPV prevention interventions is to identify these latent constructs, and the actual drivers of IPV, and seek to impact these. The other measured variables are important (as they can help capture change), but are not what we seek to impact on ultimately.

To achieve greater clarity on the drivers of IPV and the underlying latent constructs we need in-depth, rigorous qualitative research. Long-term ethnographic research has the potential to show how a confluence of different aspects of people’s lives, identities, influences, and immediate contextual factors can result in violence. This should enable a better understanding of the nature of interventions needed to prevent violence and it can also result in empirically testable hypotheses about the mechanisms through which risk factors lead to IPV.

High-quality qualitative research is also important as formative research for intervention development or adaptation in new settings. Understanding which drivers are particularly prominent, and how they operate together to increase IPV in a particular location, enables interventions to better resonate with local settings and be more effective. Building support for further qualitative research supported by funders or government will only be possible by qualitative research shifting away from descriptive analyses towards theoretically located analyses, recognizing positives and negatives of approaches, and contributing to wider debates.

Quantitative research needs to move from cross-sectional models of ‘risk factors for IPV’ to focus on developing theoretically driven models, which can test hypotheses about the drivers of, and the pathways to, IPV. Measures need to be carefully designed and included in research to enable these analyses and modeling strategies such as structural equation modelling, latent class analysis, multiple mediation and longitudinal analyses used, to start to tease apart these mechanisms and deepen our understanding. This will require large-scale longitudinal data sets, with sufficient measures, which capture the complexity of people’s lives. It may also require additional qualitative work to ensure that the measures of IPV that we use are adequately capturing women’s experiences adequately and in ways that resonate with their experiences.

The argument presented in the paper also suggests that the ecological model [5] provides an important framework to understand the multi-level nature of IPV, but does not adequately capture the interconnections between different levels, nor provide clarity about which risk-factors may be indicators of broad underlying latent constructs. In this respect it was notable in *What Works* that whilst many interventions operated at multiple levels of the ecological model (e.g. community and individual), this did not particularly predict their success. Rather, many of the most effective interventions only operated at one ecological level, but addressed multiple drivers of violence [84].

There remain a number of key questions about how drivers operate for particularly vulnerable groups, and the pathways through which this occurs. This is particularly the case for women and girls with disabilities and adolescent girls and young women. Similarly, for women and girls exposed to conflict. All these groups face much higher rates of IPV, and understanding why this is the case remains a critical challenge.

Another key question is understanding the drivers of IPV in settings where IPV rates are much higher than national averages. Is this simply an intensification of known drivers, as is often assumed [43]? Or, are there particular dynamics between drivers, or unknown drivers that are key in these processes? Understanding this is critical for identifying effective interventions and entry points for these populations, which has often eluded the field [85].

The field of violence against women and girls has developed substantially in the past six to ten years, with stronger understandings of the underlying drivers of and risk factors for IPV, which has also led to more effective IPV prevention interventions. This work needs to continue, to enable the development of interventions which are more effective to achieve the goals set out in the SDGs to eliminate violence against women and girls, including achieving gender equality.

## Paper context

There has been a rapid expansion in research on the drivers of intimate partner violence (IPV). This article provides a comprehensive review of this evidence and identifies drivers of IPV, and the causal paths through which drivers operate. It outlines the future directions of research around drivers of IPV, for more effective prevention interventions.
